# Supplementary material for: Suffering in silence: Accessing mental health care and repetitive transcranial magnetic stimulation (rTMS) for peripartum depression - A qualitative study
Source: PLoS One. 2025 Apr 24;20(4):e0321813. doi: 10.1371/journal.pone.0321813 (PMC12021197; doi:10.1371/journal.pone.0321813)
Supplement: Attachment — Submitted filename: Comments to Authors.pdf [file pone.0321813.s002.pdf]

Comments to Authors

This qualitative study explores barriers and facilitators in accessing mental health care and specifically the use of repetitive transcranial magnetic stimulation (rTMS) for peripartum depression (PPD). Through interviews with individuals experiencing PPD and health providers, it identifies systemic and personal challenges and assesses attitudes towards rTMS as an emerging treatment. I have some comments as follows.

Introduction

1. "only a proportion of mothers develop PPD". Please clearly state what percentage (proportion) this is.

Method

1. Please elaborate on the theoretical basis behind the interview design and the selection of specific questions, which can add rigor to the research method.
2. Please provide additional background information on the sampling strategy and selection criteria to clarify why these participants are representative of the broader PPD population. Otherwise, discussing the relevance and potential limitations of the sample can increase the transparency of the study.

Result

1. Overall, I feel that the paragraphs are too lengthy and contain too many quotations (narrative parts in italics). These quotations could perhaps be moved to supplementary materials or be condensed, such as by using tables or figures for presentation. This could aid readers in grasping the data more readily.

Discussion

1. Sample limited to Canada, potentially restricting generalizability to other healthcare contexts.
2. Self-selection bias may influence results, as participants interested in mental health might be more willing to share experiences.
3. Lack of longitudinal follow-up on participants' subsequent treatment decisions, limiting understanding of rTMS's long-term acceptance or impact.
